# Effects of Gui Zhi Ma Huang Ge Ban Tang on the TLR7 Pathway in Influenza Virus Infected Mouse Lungs in a Cold Environment

**DOI:** 10.1155/2018/5939720

**Published:** 2018-04-23

**Authors:** Hong-Qiong Qin, Shan-Shan Shi, Ying-Jie Fu, Yu-Qi Yan, Sha Wu, Xiao-Long Tang, Xiao-Yin Chen, Guang-Hui Hou, Zhen-You Jiang

**Affiliations:** ^1^Department of Microbiology and Immunology, School of Medicine, Jinan University, Guangzhou 510632, China; ^2^Medical College, Anhui University of Science & Technology, Huainan 232001, China; ^3^Department of Traditional Chinese Medicine, School of Medicine, Jinan University, Guangzhou 510632, China; ^4^Department of Ophthalmic Center, People's Hospital of Zhuhai City, Affiliated Hospital of Zhuhai Medical College, Jinan University, Zhuhai 519000, China

## Abstract

**Objective:**

We wished to investigate the effects of the traditional Chinese medicine Gui Zhi Ma Huang Ge Ban Tang on controlling influenza A virus (IAV) infection and improving inflammation in mouse lungs.

**Method:**

Mice were maintained in normal and cold environments and infected with IAV by intranasal application, respectively. Real-time quantitative polymerase chain reaction was used to measure mRNA expression of TLR7, myeloid differentiation primary response 88 (MyD88), and nuclear factor-kappa B (NF-*κ*B)p65 in the TLR7 signaling pathway and virus replication in lungs. Western blotting was used to measure expression levels of TLR7, MyD88, and NF-*κ*B p65 proteins. Flow cytometry was used to detect the proportion of T-helper (Th)1/Th2 and Th17/T-regulatory (Treg) cells.

**Results:**

Application of Gui Zhi Ma Huang Ge Ban Tang in influenza-infected mice in a cold environment showed (i) downregulation of TLR7, MyD88, and NF-*κ*Bp65; (ii) inhibition of transcriptional activities of promoters coding for TLR7, MyD88, and NF-*κ*Bp65; (iii) reduction in the proportion of Th1/Th2 and Th17/Treg cells.

**Conclusions:**

Gui Zhi Ma Huang Ge Ban Tang had a good therapeutic effect on mice infected with IAV, especially in the cold environment. It could reduce lung inflammation in mice significantly and elicit an anti-influenza effect by downregulating expression of the key factors in TLR7 signaling pathway.

## 1. Introduction

The influenza A virus (IAV) is an important pathogen in the respiratory tract that causes seasonal epidemics and pandemics of considerable morbidity and mortality [1, 2] IAV infection induces the host's natural immune response [3] and activation of a Toll-like receptor- (TLR-) mediated antiviral signaling pathway. The TLR family plays an important part in pathogen recognition and activation of innate immunity [4]. TLR7 is a member of the TLR family and detects single-stranded RNA (ssRNA) [5].

Currently, ribavirin [6] and oseltamivir [7, 8] are effective in suppressing viral replication in IAV infection, but the IAV is susceptible to variation. The emergence and spread of adamantane resistance and neuraminidase inhibitors (NAI) resistance would limit therapeutic options. Vaccination is known to be an effective method to prevent influenza. However, current vaccines are not highly protective if antigenically different new strains emerge, such as the outbreak of the pandemic (H1N1) 2009 virus [9, 10]. Therefore, finding a measure that protects against the emergence of an unexpected influenza strain is highly desirable.

Many Chinese herbs are used to treat and prevent influenza [11]. Compared with Western medicine, Traditional Chinese medicines (TCM) have multiple targets against different types of influenza viruses. Yinqiaosan is the representative solution of xinliangjiebiao, a medicine that can relieve fever and detoxify. Yinqiaosan is used for treating viral infectious diseases such as influenza, acute tonsillitis, pharyngitis, measles, and mumps. Xinjiaxiangruyin is a representative medicine of qushijiebiao, which has the effect of relieving “heat” and “dampness,” and is used mainly to treat summer colds. This recipe has a very good therapeutic effect for damp-heating flu. Gui Zhi Ma Huang Ge Ban Tang is a representative medicine of xinwenjiebiao with anti-influenza virus and anti-inflammatory effects. It also has antipyretic, analgesic, and other pharmacologic effects and has been used to treat colds, respiratory-tract infections, and allergies. However, studies focusing on the antivirus and anti-inflammatory mechanisms of Gui Zhi Ma Huang Ge Ban Tang are lacking.

Wan and colleagues [12] demonstrated that baicalin inhibits TLR7/MYD88 signaling pathway activation to suppress lung inflammation in mice infected with influenza A virus. Hence, using yinqiaosan and xinjiaxiangruyin as controls, we investigated the effects of Gui Zhi Ma Huang Ge Ban Tang on the TLR7 signaling pathway and on the immune balance of T cells in mouse lungs infected with the IAV in a cold environment.

## 2. Materials and Methods

### 2.1. Animals and Grouping

Experiments were undertaken under the supervision and assessment of the Laboratory Animal Ethics Committee of Jinan University (Jinan University, Guangzhou, China). All experimental procedures were carried out in accordance with the* Guide for the Care and Use of Laboratory Animals* (ninth edition; National Institutes of Health, Bethesda, MD, USA) and were approved by the Animal Ethics Committee of Jinan University.

Female specific pathogen-free (SPF) C57BL/6j mice (20 ± 1 g) were purchased from the Guangdong Experimental Animal Center (Guangdong, China). TLR7 knockout (TLR7^−/−^) mice were obtained from Taconic Farms (Germantown, NY, USA). TLR7^−/−^ mice were over 10 generations.

Thirty-six female SPF C57BL/6j mice (6°C, wild type (WT)) and 36 female SPF TLR7^−/−^mice (6°C, TLR7^−/−^) were maintained in a cold room (6 ± 1°C) with humidity of 50% and light intensity of 3000 Lx and 0 Lx under a 12-h light–dark cycle. A control group of 36 female SPF C57BL/6j mice (20°C, WT) were maintained in a normal environment (20 ± 1°C; humidity of 50%  ± 5%; light intensity of 3000 Lx and 0 Lx under a 12-h light–dark cycle).

Mice were divided randomly into six groups with six animals in each group: normal control; IAV control (“virus control”); IAV + oseltamivir (“oseltamivir”); IAV + yinqiaosan (“yinqiaosan”); IAV + Gui Zhi Ma Huang Ge Ban Tang (“Gui Zhi Ma Huang Ge Ban Tang”); IAV + xinjiaxiangruyin (“Xinjiaxiangruyin”).

### 2.2. IAV and Drugs

IAV [FM1/1/47 strain (mouse adapted)] was used for all experiments and grown in the allantoic cavities of 10-11-day-old fertile chicken eggs for 2 days at 35°C. Yinqiaosan, Gui Zhi Ma Huang Ge Ban Tang, and xinjiaxiangruyin were purchased from the First Affiliated Hospital of Jinan University.

Yinqiaosan [Detailed Analysis of Epidemic Warm Diseases, Tang Wu, 1798] contained* Forsythia* (15 g), honeysuckle (15 g), Campanulaceae (9 g), mint (9 g), bamboo leaves (6 g), raw licorice (5 g),* Nepeta* (6 g), dandouchi (6 g), burdock (6 g), and reed rhizome (10 g).

Gui Zhi Ma Huang Ge Ban Tang [Typhoid miscellaneous diseases theory, Zhong-Jing Zhang, about 219] contained Ephedra Tang [Ephedra (9 g), Guizhi (6 g), almonds (9 g), and Zhigancao (6 g)] and Guizhi Tang [Guizhi (9 g), peony (9 g), licorice (6 g), ginger (9 g), and jujube (10 g)].

Xinjiaxiangruyin [Detailed Analysis of Epidemic Warm Diseases, Tang Wu, 1798] contained* Elsholtzia* (6 g), honeysuckle (9 g), lentils (9 g),* Magnolia* (6 g), and* Forsythia* (6 g).

Oseltamivir phosphate (75 mg) was obtained from Yichang Changjiang Pharmaceuticals (Hubei, China).

### 2.3. Creation of an Influenza Model

TLR7^−/−^ mice and WT C57BL/6j mice were infected with the IAV (FM1 strain) on day 11. Mice in the virus control, oseltamivir, yinqiaosan, Gui Zhi Ma Huang Ge Ban Tang, and xinjiaxiangruyin groups were anesthetized using chloral hydrate and then infected by intranasal application of 40% LD50 (Half the lethal dose). This procedure leads to infection in the upper and lower respiratory tracts. Mice in the normal control group were anesthetized with chloral hydrate and then given an identical amount of distilled water. The physical status and weight of mice were recorded daily.

### 2.4. Anti-IAV Experiments

**Figure 1:**
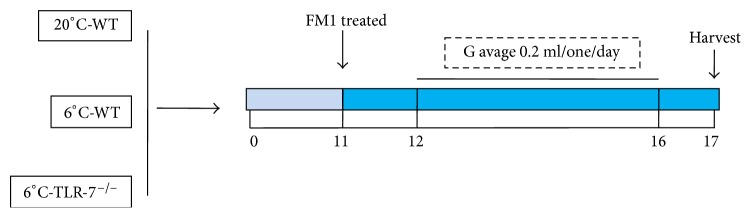
Influenza model in mice.

The drug dose was calculated based on body weight differences between humans and mice. The value for yinqiaosan was 1.12 g/mL body weight, 0.940 g/mL for Gui Zhi Ma Huang Ge Ban Tang, 0.465 g/mL for xinjiaxiangruyin, and 3 mg/mL for oseltamivir, respectively. Mice in the normal control group and virus control group were given physiologic (0.9%) saline instead. The corresponding drug concentration (or 0.9% saline) was administered (0.2 mL/day, i.g.) to each mouse 24 h after virus challenge for 5 days (day 12 to day 16) (Figure 1).

### 2.5. Tissue Harvesting

Six days after infection (day 17), after documenting the body weight, mice were anesthetized and sacrificed. The spleen and lung were harvested. The lung weight was obtained. Then, the lung weight/body weight percentage was calculated.

### 2.6. Histopathology

Lung tissue was fixed with 4% paraformaldehyde and then trimmed by a scalpel. The appropriate lung portion was selected and rinsed, dehydrated, and treated by a transparent agent xylene, immersing. After the sections had been dried and dewaxed, they were stained with hematoxylin and eosin. The tissues were sectioned, and the organization of tissue was observed at ×100 magnification.

### 2.7. Quantitative Real-Time Reverse Transcription-Polymerase Chain Reaction (RT-qPCR) of TLR7, MyD88, and NF-*κ*B p65 mRNA and Relative Replication of IAV in Lung Tissue

Total RNA was extracted with RNAiso Plus according to manufacturer (TaKaRa Bio, Shiga, Japan) instructions. cDNA synthesis and real-time PCRs were carried out using a CFX Connect Real-Time PCR Detection system (Bio-Rad Laboratories, Hercules, CA, USA) with PrimeScript™ RT Reagent kits and SYBR Premix EX Taq II according to manufacturer (TaKaRa Bio) instructions. Primers were synthesized by Generay Biotech (Shanghai, China). All primers for RT-qPCR are presented in Table 1.

After RT-qPCR, the corresponding relative mRNA expression was normalized to that of glyceraldehyde 3-phosphate dehydrogenase (GAPDH) and was calculated using the 2^−ΔΔCq^ method. Each sample was measured thrice and averaged. qPCR results were representative of three independent experiments. Gene expression levels in the virus control, oseltamivir, yinqiaosan, Gui Zhi Ma Huang Ge Ban Tang, and xinjiaxiangruyin groups were expressed relative to that of the normal control group.

### 2.8. Western Blotting for TLR7, MyD88, and NF-*κ*B p65: Western Blotting Was Performed According to Standard Procedures

Proteins were extracted from lung tissues and quantified using a bicinchoninic acid protein assay kit (MultiSciences Biotech, Hangzhou, China). An equal amount of protein (40 *μ*g/lane) was fractionated using an electrophoresis system (Bio-Rad Laboratories) on 10% polyacrylamide gels and then transferred to polyvinylidene fluoride (PVDF) membranes (Millipore, Billerica, MA, USA). PVDF membranes were blocked with 5% nonfat milk and incubated with TLR7 (1 : 1000 dilution; Cell Signaling Technology, Danvers, MA, USA), MyD88 (1 : 1000; Cell Signaling Technology), or NF-*κ*B p65 (1 : 1000; Cell Signaling Technology) in 5% BSA-TBST buffer overnight at 4°C. The loading control was GAPDH (1 : 1000; Cell Signaling Technology). After incubation with the secondary antibody (horseradish peroxidase-conjugated goat anti-rabbit IgG; 1 : 5000; MultiSciences Biotech), protein bands were detected using an electrochemiluminescence kit according to manufacturer instructions and analyzed using ImageJ software (NIH).

### 2.9. Immunofluorescence Labeling and Flow Cytometry

Peripheral blood mononuclear cells (PBMCs) were isolated from the spleen by lymphocyte separation medium according to manufacturer (MultiSciences Biotech) instructions. Different subsets of T cells were evaluated by flow cytometry. All anti-mouse-specific antibodies used were obtained from eBioscience (San Diego, CA, USA). PBMCs were stimulated with phorbol myristate acetate (12.5 *μ*g/mL; MultiSciences Biotech) and ionomycin (0.25 mg/mL; MultiSciences Biotech) in the presence of an FC receptor blocker (MultiSciences Biotech) for 5 h. PBMCs were washed and then fixed/permeabilized in fixation/permeabilization buffers (eBioscience) and stained with antibodies against cluster of differentiation (CD)4-Phycoerythrin (PE), CD25-Allophycocyanin (APC), interleukin (IL)-4-APC, interferon (IFN) gamma-biotin, IL-17A-Alexa Fluor 488, and forkhead box P3 (Foxp3)-PE-cyanine5.5. Appropriate isotype controls were used. Flow cytometry was undertaken on a FACSVerse flow cytometer (Becton Dickinson Biosciences, Franklin Lakes, NJ, USA) and analyzed using FlowJo v10 (FlowJo, Ashland, OR, USA).

### 2.10. Statistical Analyses

Statistical analyses were carried out using SPSS v22 (IBM, Armonk, NY, USA). Results are the mean ± SD. Data were analyzed using ANOVA or Student's* t*-test. *p* < 0.05 was considered significant, and *p* < 0.01 was considered to be statistically significant.

## 3. Results

### 3.1. Physical Status of Mice in Each Group

Eighteen hours after the induction of infection, in addition to the normal control group, mice showed varying degrees of influenza symptoms: lying down in a curled up position, dyspnea, being apathetic, loss of luster of body hair, and reduced food intake. During the administration period, influenza symptoms in the oseltamivir group were mild, and mice in the yinqiaosan and Gui Zhi Ma Huang Ge Ban Tang groups had good physical status in the normal environment. In the cold environment, compared with the virus control group, the physical status of mice in the Gui Zhi Ma Huang Ge Ban Tang group was better.

### 3.2. Changes in Body Weight

The percentage loss in body weight was calculated using the following formula: Percentage loss in body weight = [(bodyweight at day 11 – body weight at day 17)/bodyweight at day 11] × 100%.

**Figure 2:**
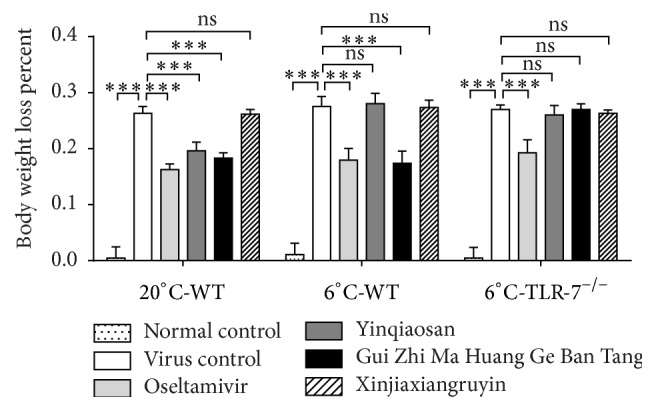
Changes in body weight of mice. The body weight of mice in virus control, oseltamivir, yinqiaosan, Gui Zhi Ma Huang Ge Ban Tang, and xinjiaxiangruyin groups, ^*∗∗∗*^*p* < 0.001, ns, *p* > 0.05.

The body weight changes of mice in the experimental groups are shown in Figure 2. Body weight of mice in the normal control group showed no changes. After IAV infection, except for the normal control group, the physical status of the mice in other groups started to deteriorate, and they began to lose body weight. The weight of mice in the virus control group decreased more quickly. There were no significant differences (*p* > 0.05) in body weight between the oseltamivir, yinqiaosan, and Gui Zhi Ma Huang Ge Ban Tang groups in the normal environment. In the cold environment, the oseltamivir group and Gui Zhi Ma Huang Ge Ban Tang group had higher body weight. There were no significant differences (*p* > 0.05) between the virus control group and xinjiaxiangruyin, yinqiaosan, and Gui Zhi Ma Huang Ge Ban Tang groups of TLR7^−/−^ mice.

### 3.3. Changes in Lung Weight/Body Weight

**Figure 3:**
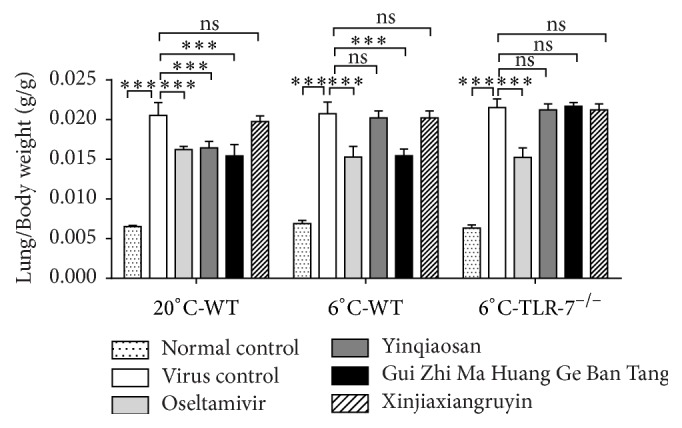
Changes in lung weight/body weight of mice. ^*∗∗∗*^*p* < 0.001, ns, *p* > 0.05.

Changes in the lung weight/body weight of mice in the different experimental groups are shown in Figure 3. The lung weight/body weight of mice in the virus control, oseltamivir, and xinjiaxiangruyin, yinqiaosan, and Gui Zhi Ma Huang Ge Ban Tang groups increased compared with that in the normal control group. In the normal environment, the lung weight/body weight of mice in the virus control group increased more than that in the oseltamivir, yinqiaosan, and Gui Zhi Ma Huang Ge Ban Tang groups. In the cold environment, the lung weight/body weight of mice in the virus control group increased more than that in the oseltamivir and Gui Zhi Ma Huang Ge Ban Tang groups. There were no significant differences (*p* > 0.05) between the virus control group and the oseltamivir, yinqiaosan, and Gui Zhi Ma Huang Ge Ban Tang groups of TLR7^−/−^ mice.

### 3.4. Morphologic Changes in Lung Tissue

**Figure 4:**
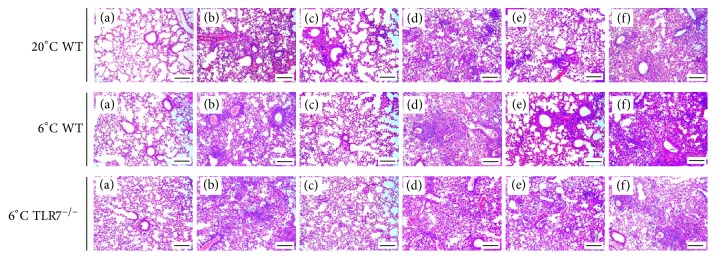
Effects of yinqiaosan, Gui Zhi Ma Huang Ge Ban Tang, and xinjiaxiangruyin on histology in mice. (A-F) Representative staining (hematoxylin and eosin) of histologic sections of all groups at day 17, (A) normal control, (B) virus control, (C) oseltamivir, (D) yinqiaosan, (E) Gui Zhi Ma Huang Ge Ban Tang, and (F) xinjiaxiangruyin. All images were obtained at ×200 magnification, scale bar = 100 *μ*m.

The integrity of the structure of alveoli before IAV infection was shown by examination of pathologic tissue sections (Figure 4(a)). After IAV infection, diffuse damage was seen in the alveoli, alveolar sacs, alveolar tubes, alveolar septa, and bronchi. There was considerable lymphocyte infiltration in the pulmonary interstitium (Figure 4(b)). Compared with the virus control group, WT mice in yinqiaosan and Gui Zhi Ma Huang Ge Ban Tang groups in the normal environment and WT mice in the Gui Zhi Ma Huang Ge Ban Tang group in the cold environment showed a clear alveolar structure, with only a small number of inflammatory cells. In the TLR7^−/−^ mice of xinjiaxiangruyin, yinqiaosan, and Gui Zhi Ma Huang Ge Ban Tang groups, there was vascular congestion, alveolar expansion, and pulmonary interstitial edema, and alveolar septa were filled with inflammatory cells. There were no obvious differences between the virus control group and xinjiaxiangruyin, yinqiaosan, and Gui Zhi Ma Huang Ge Ban Tang groups in TLR7^−/−^ mice.

### 3.5. Changes in IAV Replication in the Lungs

**Figure 5:**
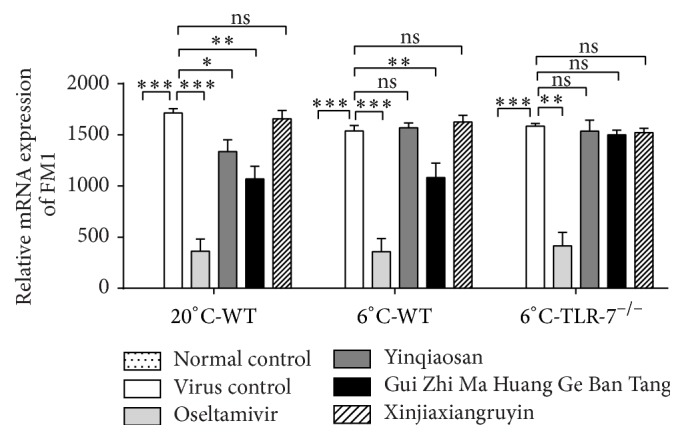
Effect of yinqiaosan, Gui Zhi Ma Huang Ge Ban Tang, and oseltamivir on IAV (FM1) replication. ^*∗*^*p* < 0.05, ^*∗∗*^*p* < 0.01, ^*∗∗∗*^*p* < 0.001, ns, *p* > 0.05.

There was no IAV replication in the normal control group. IAV-infected mice in the virus control group had significantly greater replication than that in the normal control group (*p* < 0.001). Oseltamivir reduced virus replication significantly compared with the virus control group (*p* < 0.001). In the normal environment, IAV replication in the yinqiaosan and Gui Zhi Ma Huang Ge Ban Tang groups was significantly different compared with that in the oseltamivir group (*p* < 0.05), but there were no significant differences between the virus control group and xinjiaxiangruyin group (*p* > 0.05). In the cold environment, IAV replication in the Gui Zhi Ma Huang Ge Ban Tang group was significantly higher than that in the oseltamivir group (*p* < 0.05), but there were no significant differences between the virus control group and yinqiaosan and xinjiaxiangruyin groups (*p* > 0.05). There were no significant differences (*p* > 0.05) between the virus control group and xinjiaxiangruyin, yinqiaosan, and Gui Zhi Ma Huang Ge Ban Tang groups of TLR7^−/−^ mice (Figure 5).

### 3.6. mRNA Expression Levels of TLR7, MyD88, and NF-*κ*B

**Figure 6:**
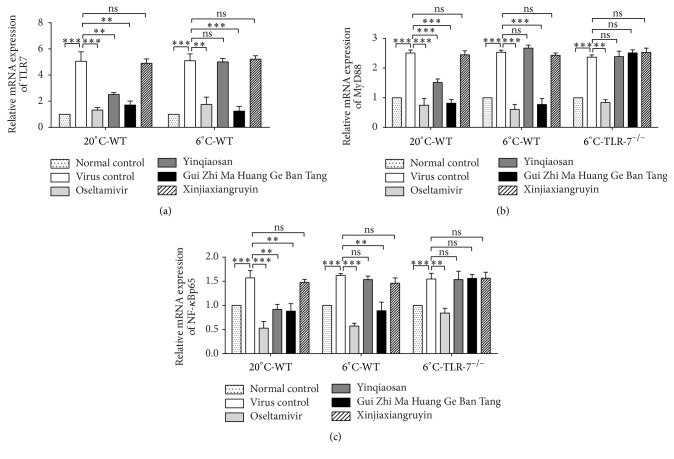
mRNA expression of TLR7 (a), NF-*κ*B p65 (b), and MyD88 (c) in the mouse lung tissue. ^*∗∗*^*p* < 0.01, ^*∗∗∗*^*p* < 0.001, ns, *p* > 0.05.

Expression of the mRNA of TLR7 (A), MyD88 (B), and NF-*κ*B (C) via the TLR7 signaling pathway in pulmonary immunocytes was increased significantly in the virus control group (*p* < 0.001) (Figure 6). In the normal environment, treatment with oseltamivir, yinqiaosan, or Gui Zhi Ma Huang Ge Ban Tang reduced mRNA expression in the TLR7 signaling pathway. Compared with the virus control group, mRNA expression in the TLR7 signaling pathway was downregulated in the oseltamivir, yinqiaosan, and Gui Zhi Ma Huang Ge Ban Tang groups (*p* < 0.05). In the cold environment, compared with the virus control group, the relative mRNA expression of TLR7, MyD88, and NF-*κ*B p65 in the oseltamivir and Gui Zhi Ma Huang Ge Ban Tang groups was downregulated (*p* < 0.05). There were no significant differences between the virus control and the yinqiaosan and xinjiaxiangruyin groups (*p* > 0.05) (Figure 6).

### 3.7. Relative Protein Expression of TLR7, MyD88, and NF-*κ*B p65

**Figure 7:**
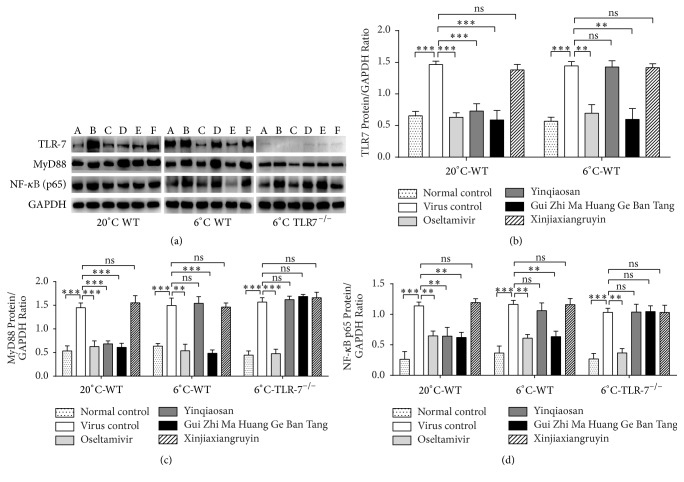
Relative protein expression of TLR7, NF-*κ*B p65, and MyD88 (a): A: normal control, B: virus control; C: oseltamivir, D: yinqiaosan, E: Gui Zhi Ma Huang Ge Ban Tang, and F: xinjiaxiangruyin. Protein expression of TLR7 (b), MyD88 (c), and NF-*κ*B p65 (d). ^*∗∗*^*p* < 0.01, ^*∗∗∗*^*p* < 0.001, ns, *p* > 0.05.

Western blotting revealed that expressions of protein levels of TLR7 (A), MyD88 (B), and NF-*κ*B p65 (C) were enhanced gradually in the virus control group compared with the normal control group. Compared with the virus control group, the relative protein expression of TLR7, MyD88, and NF-B p65 was downregulated in the oseltamivir, yinqiaosan, and Gui Zhi Ma Huang Ge Ban Tang groups in the normal environment. In the cold environment, compared with the virus control group, the relative protein expression of TLR7, MyD88, and NF-*κ*B p65 of the oseltamivir and Gui Zhi Ma Huang Ge Ban Tang groups was downregulated (*p* < 0.05). There were no significant differences between the virus control group and yinqiaosan and xinjiaxiangruyin groups (*p* > 0.05). Western blotting results were in accordance with RT-qPCR results (Figure 7).

### 3.8. Detection of Th1, Th2, Th17, and T-Regulatory (Treg) Cells

**Figure 8:**
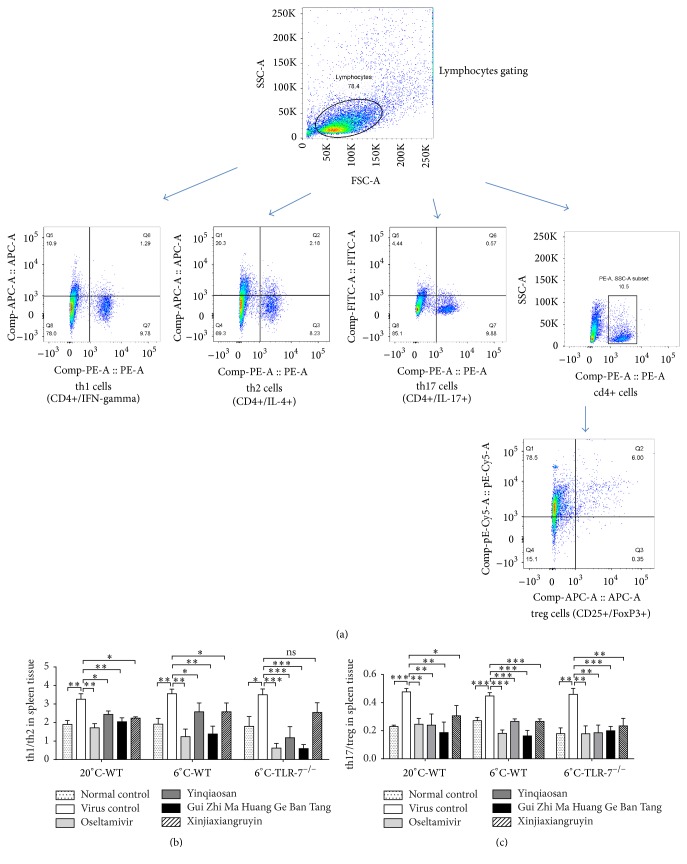
Flow cytometry of CD4+ T-cell subsets (a). Changes in the proportions of Th1/Th2 cells (b) and Th17/Treg cells (c). ^*∗*^*p* < 0.05, ^*∗∗*^*p* < 0.01, ^*∗∗∗*^*p* < 0.001, ns, *p* > 0.05.

Flow cytometry was used to detect the proportion of the Th1/Th2 and Th17/Treg cells. These proportions increased after the induction of infection. Th1/Th2 cells differentiated towards Th1 cells, and Th17/Treg cells differentiated towards Th17 cells. IAV-infected mice in the oseltamivir as well as the xinjiaxiangruyin, yinqiaosan, and Gui Zhi Ma Huang Ge Ban Tang groups had lower proportions of these T-cell subsets than the IAV-infected mice of the virus control group. The T cells from the xinjiaxiangruyin, yinqiaosan, and Gui Zhi Ma Huang Ge Ban Tang groups differentiated into Th1 or Th17 cells, and the proinflammatory effects of T cells were inhibited (Figure 8).

## 4. Discussion

Humans may be infected by the influenza virus at any time of the year, but infection happens more likely in winter. In addition, most people are indoors during winter and the windows are often closed, resulting in poor air circulation which increases the chance of the virus spreading.

In the present study, compared with yinqiaosan and xinjiaxiangruyin, Gui Zhi Ma Huang Ge Ban Tang had a good therapeutic effect on mice infected with the IAV (FM1 strain), especially in the cold environment. Gui Zhi Ma Huang Ge Ban Tang could reduce lung inflammation in mice significantly and elicit an anti-influenza effect by downregulating expression of the key factors of the TLR7 signaling pathway.

TLR7 [12, 13] is an important member of the TLR family. TLRs can identify the pathogen-related molecular patterns of various microbes, including bacteria, viruses, protozoa, and fungi, and have an important role in natural immunity. TLRs can induce the expression of IFN-*α*, IL-12, and other cytokines by identifying ssRNA. IAV infection triggers a series of immune responses that can limit viral replication and viral infection.

The cells that express TLR7 are mainly immunocytes such as plasmacytoid dendritic cells and macrophages. These immunocytes can recognize viral ssRNA through TLR7 and elicit an antiviral immune response through the MyD88 pathway, which leads to activation of costimulatory molecules and cytokine production followed by a specific immune response. If activation of this pathway is dysfunctional, it leads to expression of many proinflammatory mediators, resulting in inflammation [12, 14, 15]. TLR7 has been shown to mediate multiple viral immune responses [16–18], including those to the human immunodeficiency virus-1, influenza virus, vesicular stomatitis virus, and hepatitis-C virus.

The present study showed that xinjiaxiangruyin, yinqiaosan, and Gui Zhi Ma Huang Ge Ban Tang could lower the ratio of Th1/Th2 cells and Th17/Treg cells in normal and cold environments, as well as in TLR7^−/−^ mice, but Gui Zhi Ma Huang Ge Ban Tang is better. Th1, Th2, Th17, and Treg cells are different subpopulations differentiated by CD4+ T cells and have different roles in the immune response [19–22]. Th1 cells mainly secrete IL-2, IFN-*γ* and other cytokines and are mainly involved in cellular immune responses, mediating cytotoxic delayed-type hypersensitivity and local inflammation. They can also inhibit the formation of Th2 cells. Th2 cells mainly secrete IL-4 and IL-6 and are associated with humoral immunity, stimulating the activation and proliferation of B cells, and antibody secretion. Th17 cells can secrete IL-17, IL-21, and other cytokines. IL-17 is a strong and effective proinflammatory cytokine [22, 23] and can stimulate the secretion of granulocyte-colony stimulating factor and prostaglandin (PG)E2; enhance the function of IL-1 and tumor necrosis factor (TNF); induce the secretion of NF-*κ*B, IL-6, IL-8, and other factors in promoting the inflammatory response and play an important part in autoimmune injury [24, 25]. Treg cells secrete TGF-*β* and IL-10, which inhibit the differentiation and function of Th17 cells. In the infection process, especially in later stages, Treg cells can regulate and control inflammatory reactions to prevent autoimmune injury.

In healthy individuals, the ratios of Th1/Th2 cells and Th17/Treg cells [26, 27] are in a dynamic balance. We found that IAV (FM1 strain) infection could activate the immune system, increase the ratios of Th1/Th2 cells and Th17/Treg cells, and lead to an increased proinflammatory response. The ratios of Th1/Th2 cells and Th17/Treg cells in the virus control group were significantly higher than those in the normal control group, suggesting that IAV infection could promote the differentiation of CD4+ T cells to Th1 cells and Th17 cells.

TCM has been employed for thousands of years [28]. Studies have shown that Gui Zhi Ma Huang Ge Ban Tang [29] has effects on infection of the upper respiratory tract, pneumonia, fever, urticaria, allergic purpura, and severe psoriasis. Guizhi [30] and Ephedra have anti-inflammatory, antiviral, antitumor, antibacterial, and immunoregulatory effects. Ginger [31] has anti-inflammatory, antimicrobial, and antioxidant effects. Ginger contains gingerol, which can inhibit production of nitric oxide, IL-6, and TNF in glial cells. Peony [32], Guizhi [33], and ginger have anti-inflammatory effects and can reduce expression of PGE2, NF-*κ*B, and other mediators of the inflammatory response. The chemical constituents in Gui-Zhi-Tang [34] were studied by rapid resolution liquid chromatography quadrupole time-of-flight mass spectrometry combined with rapid resolution liquid chromatography-diode array detector-ion trap mass spectrometry, and a total number of 187 compounds were detected. 3-phenyl-propenal is one of the principle compounds isolated from Guizhi. 3-phenyl-propenal blocked the overexpression of TLR3, TLR4, and their downstream signaling components MyD88 and TRAF-6 [35]. There are apparent changes not only in quantity but also in quality between decoction and its major constituted herbs. Meanwhile any changes in composition may lead to a change in efficacy [36].

## 5. Conclusions

We demonstrated that, compared with yinqiaosan and xinjiaxiangruyin, Gui Zhi Ma Huang Ge Ban Tang had a good therapeutic effect on mice infected with IAV (FM1 strain), especially in the cold environment. Gui Zhi Ma Huang Ge Ban Tang could reduce lung inflammation in mice significantly and elicit an anti-influenza effect by downregulating expression of the key factors of the TLR7 signaling pathway.

## Figures and Tables

**Table 1:** Primers used for RT-qPCR analysis.

Gene	Primer	Sequence
GAPDH	Forward	5′-CTGAGCAAGAGAGGCCCTATCC-3′
	Reverse	5′-CTCCCTAGGCCCCTCCTGTT-3′
FM1	Forward	5′-GACCAATCCTGTCACCTCTGAC-3′
	Reverse	5′-AGGGCATTTGGACAAAGCGTCTA-3′
TLR-7	Forward	5′-GGGTCCAAAGCCAATGTG-3′
	Reverse	5′-TGTTAGATTCTCCTTCGTGATG-3′
MyD88	Forward	5′-CGATTATCTACAGAGCAAGGAATG-3′
	Reverse	5′-ATAGTGATGAACCGCAGGATAC-3′
NF-*κ*B p65	Forward	5′-ATTCTGACCTTGCCTATCTAC-3′
	Reverse	5′-TCCAGTCTCCGAGTGAAG-3′
